# Rapid updating of spatial working memory across saccades

**DOI:** 10.1038/s41598-017-18779-9

**Published:** 2018-01-18

**Authors:** Paul J. Boon, Silvia Zeni, Jan Theeuwes, Artem V. Belopolsky

**Affiliations:** 1 Department of Experimental and Applied Psychology, Vrije Universiteit, Amsterdam, The Netherlands; 2 School of Psychology, University of Nottingham, Nottingham, UK

## Abstract

Each time we make an eye movement, positions of objects on the retina change. In order to keep track of relevant objects their positions have to be updated. The situation becomes even more complex if the object is no longer present in the world and has to be held in memory. In the present study, we used saccadic curvature to investigate the time-course of updating a memorized location across saccades. Previous studies have shown that a memorized location competes with a saccade target for selection on the oculomotor map, which leads to saccades curving away from it. In our study participants performed a sequence of two saccades while keeping a location in memory. The trajectory of the second saccade was used to measure *when* the memorized location was updated after the first saccade. The results showed that the memorized location was rapidly updated with the eyes curving away from its spatial coordinates within 130 ms after the first eye movement. The time-course of updating was comparable to the updating of an exogenously attended location, and depended on how well the location was memorized.

## Introduction

Saccadic eye movements ensure that the brain gets relevant information from the environment as quickly as possible. To achieve this, these eye movements can reach peak velocities of 750°/s. As a result, each time we make a saccade, positions of objects on the retina change quickly and dramatically. Given that the oculomotor system is retinotopically organized (eye-centered), the locations of relevant objects need to be rapidly updated with each saccade. For example, while reading this text, you might reach for the cup of coffee you have just placed next to you. Although the cup stays at the same location in the world, it will occupy a different part of the retina each time the eyes move to the next word. The situation becomes even more complex if the object of interest is no longer visible, as a result of being, for example, occluded by another object. This means that the *internal*, *memorized* position of the object has to be updated with each eye movement. The current models of spatial working memory updating during eye movements suggest that this process is quite time-consuming. The time delay is assumed to be the result of the native coordinate system of memory representations which is thought to be retinotopic^1,2^. This means that the memorized locations are thought to naturally move with each eye movement and a special effort is necessary to gradually transform these retinotopic representations into the world-centered (spatiotopic) coordinates. Previous studies used perceptual discrimination tasks to investigate the updating of memorized content. There is a close link between attentional and working memory representations^3–5^. For example, memorizing a location leads to attentional facilitation at that location^6^. Therefore, enhanced processing of a probe stimulus can be used as an indication of updating of spatial working memory across saccades. Studies using this technique showed that attention resided at the retinotopic location of the remembered object almost immediately after saccade. In contrast, it took up to 400 ms before attentional facilitation was also observed at the spatiotopic location^1,7^.

The time-course of spatial working memory updating seems very slow, especially compared to the recent demonstrations of rapid updating of exogenous attentional signals^8–10^. In one study^8^, an irrelevant, but salient cue was flashed while participants were planning a saccade. The results showed that attention was maintained at the spatiotopic location of the cue before and after saccade, despite a change in the retinal location of the cue induced by a saccade. The retinotopic representation of the cue, however, decayed very rapidly after the eye landed. Importantly, right before an impending saccade, attention was allocated to the future retinotopic location of the cue. This predictive allocation of attention is thought to underlie the maintenance of exogenous attention at the spatiotopic locations across saccades^11^. Such predictive allocation of attention could be accomplished by the physiological process of remapping of receptive fields in anticipation of a saccade, transforming stimulus representation from pre-saccadic retinotopic receptive field to post-saccadic retinotopic receptive field^12^. Similar predictive and rapidly emerging spatiotopic representations have also been demonstrated for inhibition of return^13–15^ (but see ref.^16^). In addition, several recent studies show that spatiotopic memory representations remain quite accurate across saccades^17,18^. Boon and colleagues^17^ demonstrated that manipulating the reliability of the retinal signals after the saccade by shifting the saccade target had little effect on the accuracy of the memory performance, especially immediately after the saccade landing. This suggests that a spatiotopic representation is available very soon after the saccade.

Recently, Jonikaitis & Belopolsky^19^ demonstrated that spatiotopic representations also emerge rapidly in the oculomotor system. They used saccadic curvature to examine whether an attended distractor is represented in retinotopic or spatiotopic coordinates after an eye movement. This saccade curvature has been shown to reflect the allocation of spatial attention^20^ and attributed to competition for potential saccade targets in the oculomotor map, supposedly in the intermediate layers of the superior colliculus^21^. Strong target-distractor competition and failure to suppress competing distractor representations has been suggested to lead to saccade curvature towards distractor locations, whereas successful suppression of the competing distractor representations has been suggested to result in curvature away from the distractor location^22^. Furthermore, the more salient the distractor, the more competition and curvature it evokes^23^. In the study by Jonikaitis & Belopolsky^19^ participants performed a sequence consisting of a horizontal and a vertical saccade. The oculomotor competition was induced by briefly presenting a task-irrelevant distractor at different times during the sequence. Despite the intervening saccade, the second saccade curved away from the spatiotopic location of the distractor that was presented before the first saccade. Furthermore, this saccade curvature away increased with increasing the salience of the distractor. The results clearly showed that not only the information about distractor’s spatial location, but also the information about its relative salience was transferred across saccades. They concluded that in the oculomotor system spatiotopic representations emerge rapidly and automatically.

The goal of the present study was to investigate the time-course of updating memorized locations in the oculomotor system. Does updating of endogenously maintained information in the oculomotor system involve the same mechanism as updating of exogenous attention, or is this a time-consuming and effortful process? To examine this, we modeled our paradigm after Jonikaitis and Belopolsky^19^, but instead of presenting a salient distractor, participants were instructed to keep a location in memory. Previous studies have shown that this evokes persistent activity in the oculomotor map, causing the eyes to curve away from the remembered location^24–26^. The trajectory of the second saccade was used to assess whether the memorized location was represented in retinotopic or spatiotopic coordinates after the first eye movement. Crucially, we took advantage of the variability in the time interval between the two saccades to determine when the spatiotopic representation emerges. If memorized locations are rapidly updated, we expected the second saccade to curve away from the spatiotopic location even when the intersaccadic interval is short. However, if the formation of a spatiotopic representation is a slow and effortful process, saccades should only curve away from the spatiotopic location after the longer intersaccadic intervals.

## Experiment 1

In Experiment 1 participants were instructed to hold a location in memory and make a rapid saccade sequence as soon as two saccade targets appeared. Trajectories of the second saccade were used to measure how this location was represented after the first saccade. We took advantage of the natural variability in the time interval between the two saccades to determine the time course of updating. If memorized locations are rapidly updated the second saccade is expected to curve away from the spatiotopic location of the memorized item, even when the interval between both saccades is short. However, if updating is a slow and effortful process, saccades should only curve away from this location when this interval is longer.

### Methods

Eleven participants, aged between 21 and 32 (mean 25, 6 female), participated in Experiment 1, consisting of two 75 minute experimental sessions, completing on average 576 trials. All participants received either money or study credit. The present and following experiments were approved by the local ethics committee of the Vrije Universiteit Amsterdam, and were performed in accordance with the relevant guidelines and regulations. Participants received information about the study and their rights and gave a written informed consent. Participants were naïve with respect to the aim of the study and had normal or corrected to normal visual acuity. The experiments were conducted in a darkened room. Stimuli were presented on a 21 inch monitor (monitor type: Samsung 2233RZ) with a spatial resolution of 1680 × 1050 pixels and a refresh rate of 120 Hz. Participants viewed the monitor from a distance of 70 cm, and eye movements were recorded with Eyelink 1000 (SR Research), sampling at 1000 Hz. Saccades were detected using a velocity criterion of 35° s^−1^ and an acceleration threshold of 9500° s^−2^. The fixation dot and saccade targets were black open circles with a radius of 0.17° placed on a grey background with a luminance of 17 cd/m^2^. The memory cue and memory test were a small empty white box of 0.7° width and height.

**Figure 1:**
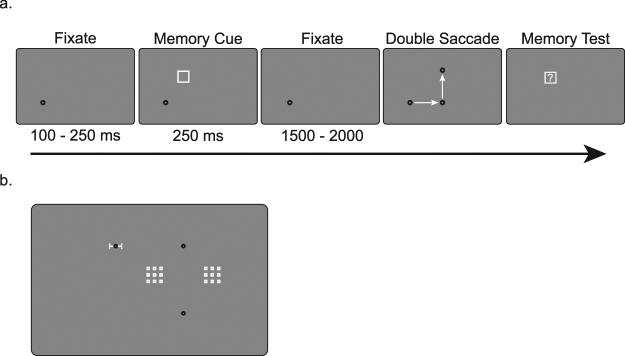
Experimental paradigm of Experiment 1. (**a**) The participants fixated the fixation dot and remembered the location of the memory cue (a white square). After a retention interval two saccade targets appeared, and participants had to make a sequence of two saccades. At the end of the trial participants had to judge whether a test stimulus was presented at the same or a slightly different location as the remembered cue. (**b**) The white squares indicate possible locations of the memory cue in a trial with a left/downward saccade sequence. The white error bar indicates possible locations of the fixation dot relative to the first saccade target.

Each trial started with a fixation dot (Fig. 1a). Depending on the direction of the first saccade, this dot was placed at 9° either left or right from the center of the screen. Depending on the direction of the second saccade this dot was placed at 5° above or below the center of the screen. To correct for eye drift participants had to fixate the fixation dot and simultaneously press the spacebar to start a trial. After a variable fixation period (100–250 ms) the memory cue appeared. Participants were instructed to hold the precise location of the cue in memory throughout the trial. After 250 ms, the memory cue was removed, and participants continued fixating. After 1500–2000 ms the saccade targets appeared. Participants made two saccades: a leftward/rightward horizontal saccade to the first saccade target, followed by an upward/downward vertical saccade to the second saccade target.

The location of this memory cue was randomly picked from nine possible locations within a 3° by 3° box (Fig. 1b). This box could be located at two different locations. In the counterclockwise condition (CCW), this box was placed at the same side as the initial fixation dot. This was done in such a way that after the first saccade its retinotopic and spatiotopic location were at an equal horizontal distance at both sides relative to the saccade targets. In the clockwise condition (CW), it was placed at the opposite side of the initial fixation dot. In this way both its retinotopic and spatiotopic location were at the same side relative to the saccade targets.

On each trial, the distance between the fixation and first saccade target was randomly varied between 9° and 11° by changing the location of the fixation dot. The distance between the first and second saccade target was always 9°. The saccade targets were visible until two saccades had been executed or 2000 ms passed without an eye movement being detected. If the direction of one of the detected saccades was more than 30° of arc away from its target, a beep sounded and the trial was discarded from the analysis. At the end of the trial participants had to judge whether a test stimulus was presented at the same or a slightly different location as the memorized cue. The distance separating the memory cue and the test stimulus was adjusted after every 10 trials to keep performance in the range of 65–85% correct. The initial distance was set at 2.5°. If memory performance was between 85% and 90% correct, the separation distance was decreased by 0.25° to make the task more challenging. If performance exceeded 90%, the distance was decreased by 0.5°. Likewise, performance between 60–65% correct or less than 60% correct increased the distance by 0.25° or 0.5°, respectively, to make the task easier. The minimum separation distance was 0.5°. At the end of each block participants received feedback about their performance.

#### Saccade curvature

For each 1-ms sample point that was further than 0.5° from the central fixation and further than 0.5° from the endpoint of the saccade the angular deviation relative to a straight line from the starting point of the saccade to the saccade endpoint was calculated. A median of these deviations was calculated for each saccade.

**Figure 2:**
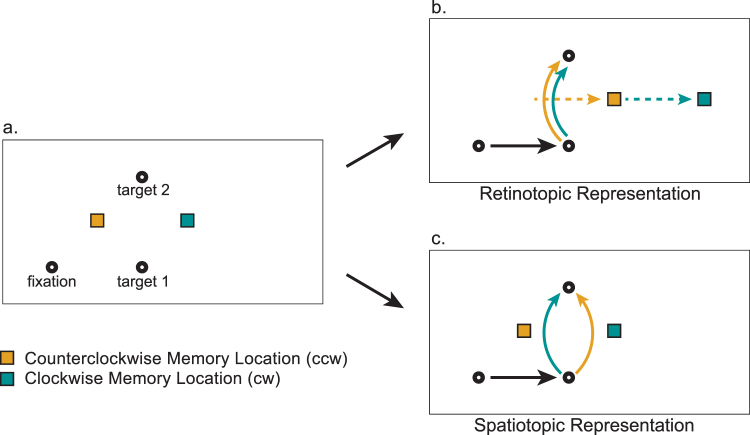
Experimental predictions. Participants performed a sequence of two saccades while holding a location in memory. The memorized location could be presented either counterclockwise (orange) or clockwise (blue) from the second saccade target (**a**). Trajectories of the second saccade were used to measure the effect of the first saccade on the representation of the remembered location. The curved lines illustrate the predicted curvature away from retinotopic (**b**) and spatiotopic locations (**c**). If the memorized location is rapidly updated, we expected the second saccade to curve away from its spatiotopic location at short intersaccadic intervals. However, if the formation of a spatiotopic representation is a slow and effortful process, saccades should only curve away from this location at longer intersaccadic intervals.

These data were further analyzed using two different methods. The first method involved binning of the data. For each participant and each condition the data were divided into four latency bins based on interval between the landing of the first saccade and the start of the second saccade. For each of these latency bins the curvature was averaged across saccade directions. Finally, the difference between curvature in the clockwise and counterclockwise condition was calculated, such that positive values indicate that saccades curved away from the spatiotopic location of the memory cue (see Fig. 2 for the experimental predictions, for a similar method see refs^24^ and^27^).

To circumvent the problem of having different latency distributions for each participant and be able to inspect the time course of updating in greater temporal detail, we smoothed saccade curvature data with weights based on a (moving) Gaussian window (σ = 20 ms), resulting in a continuous estimation of curvature for intersaccadic intervals between 100 and 275 ms. We did so for each participant and condition. Subsequently, the difference between the time series for counterclockwise and clockwise memory location was calculated, such that positive values indicate that saccades curved away from the spatiotopic location of the memory cue. For each millisecond in the smoothed time series, a weighted average across subjects was calculated. How much the curvature of a subject contributed to this average depended on the sum of the weights of the trials contributing to that single time point (see also ref.^28^).

The smoothed data was statistically tested for differences between conditions using group-level permutation testing with cluster correction for multiple comparisons. For every time-point, a paired samples t-test was used to detect differences between the clockwise and counterclockwise condition. This resulted in clusters of significant time-points. Next, for each participant, the condition labels of every trial (counterclockwise or clockwise memory location) were randomly shuffled in 1000 iterations. For each iteration and each condition, the shuffled data was smoothed and for each time-point a t test was performed on the difference between both conditions. At each permutation, the cumulative t value of each cluster of significant t values was determined, resulting in a distribution of cluster sizes under the null-hypothesis of no difference between counterclockwise and clockwise memory locations. The cluster was considered significant if its cumulative t value was equal to or exceeded the cumulative t-value corresponding to the 95th percentile of the distribution under the null-hypothesis (p < 0.05).

#### Plotting saccade trajectories

To determine the average saccade trajectories, eye position samples of each saccade were rotated so that all trials matched the rightward/upward saccade sequence. Since every saccade had different amplitude we normalized the eye positions samples for saccade amplitude. For each sample point its position relative to the total saccade amplitude was calculated. These normalized sample points were divided in ten amplitude bins. For each amplitude bin the average distance of the sample points away from the straight line connecting start and endpoint of the saccade was calculated. Subsequently, these values were averaged across trials and participants. Trajectories were plotted by connecting the values in each amplitude bin. This was done separately for each memory location (memory cue counterclockwise or clockwise) and latency bin (1–4).

Note that every person has its own idiosyncratic baseline saccade trajectory. To correct for this we calculated baseline saccade trajectory by averaging saccade trajectories for clockwise and counterclockwise memory locations. Next, we subtracted out this baseline from the trajectories for both clockwise and counterclockwise memory location. This way, if there is no difference between the trajectories in both conditions, both will be plotted as a vertical line. Any effect of the memory location will result in both lines curving to either side.

#### Memory performance

To ensure that participants were maintaining spatiotopic representations we analyzed memory performance. Because the overall accuracy on the memory test was fixed at roughly 75% by the staircase procedure, we analyzed memory test performance as measured by the average distance separating “same” and “different” test positions for each participant. A larger separation distance indicates that greater memory probe displacement was required to detect the discrepancy between the memorized and probed locations.

### Results

One participant performed poorly on the memory test, with the separation distance increasing to 8° by the end of the session, and was therefore excluded from further analysis. The other participants all performed well on the memory test, with the maximum separation distance not exceeding 3.75°.

Trials in which a saccade was detected before the onset of the saccade targets were discarded. If the first saccade was faster than 80 ms or slower than 1000 ms, or did not start within 2° of fixation dot, the trial was discarded. If the intersaccadic interval was shorter than 80 ms or longer than 1000 ms, the second saccade did not start within 3° of the first saccade target, or was shorter than 5° the trial was also discarded. This resulted in an average loss of 14% of all trials.

**Figure 3:**
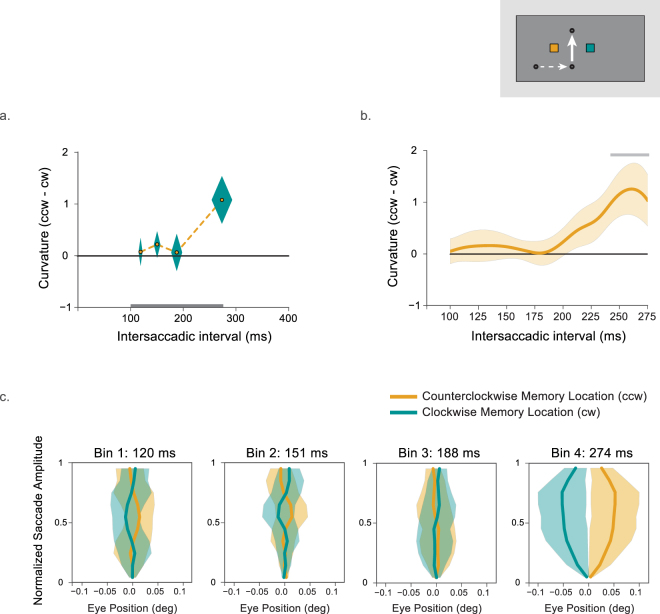
Results of Experiment 1. (**a**) Curvature difference for all four latency bins (counterclockwise minus clockwise memory location). The diamond shapes represent the within-subjects SEM calculated over curvature (vertical dimension) and the between-subjects SEM calculated over the mean intersaccadic latencies for that latency bin (horizontal). The grey line at the bottom represents the interval for which data was smoothed. (**b**) Average of the smoothed curvature of all participants (counterclockwise minus clockwise memory location). Shaded error bars indicate the within-subjects SEM calculated over curvature. The grey line indicates a cluster that did not survive the cluster-based permutation testing. (**c**). Average saccade trajectories while memorizing locations at counterclockwise (orange) and clockwise (blue, see inset) locations for the three latency bins. Error bars indicate the within-subjects SEM.

The difference in curvature between both conditions (counterclockwise minus clockwise) in each latency bin is plotted in Fig. 3a. Figure 3b shows the difference between these conditions as calculated by Gaussian smoothing. Figure 3c shows the normalized saccade trajectories in both the clockwise and counterclockwise conditions. Overall, saccades curved away from the spatiotopic location of the memorized location (counterclockwise minus clockwise = 0.42°, one-tailed t-test: t(9) = 1.99, p = 0.04, Cohen’s d = 0.63). A repeated-measures one-way ANOVA on the curvature difference (counterclockwise minus clockwise) with latency bin (1–4) as a factor revealed no significant effect of latency bin on the degree of curvature (F(3,27) = 1.99, p = 0.14). This suggests that curvature away from the spatiotopic location was statistically the same across the intersaccadic interval. Since we were interested in detailed time-course of updating, planned comparisons were conducted. There was no significant spatiotopic curvature for the shortest three latency bins (one-tailed t-tests, bin 1: t(9) = 0.29, p = 0.39, bin 2: t(9) = 0.86, p = 0.21, bin 3: t(9) = 0.19, p = 0.43), but only for the fourth latency bin (t(9) = 2.35, p = 0.022, Cohen’s d = 0.74). This suggests that the overall curvature away from the spatiotopic location was primarily driven by the longest latency bin. The smoothed curvature difference time-course revealed a similar pattern. For intersaccadic intervals shorter than 200 ms saccades did not curve away from the spatiotopic location of the memory cue. However, from around 200 ms curvature difference increased and leveled off around 250 ms. Although there was a significant cluster from 230 to 275 ms this was too small to survive the (relatively conservative) cluster-based permutation testing (p < 0.12).

**Figure 4:**
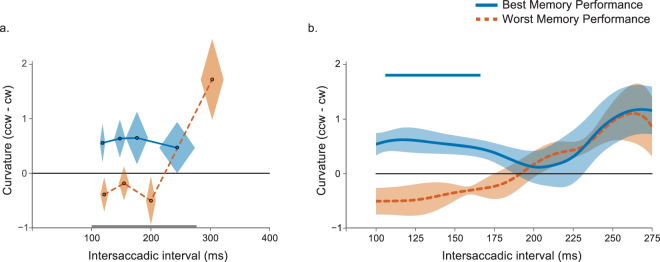
Saccade curvature as a function of memory performance in Experiment 1. (**a**) The data from the best (blue) and worst performing participants (orange) on the memory test. The diamond shapes represent the within-subjects SEM calculated over curvature (vertical dimension) and the between-subjects SEM calculated over the mean intersaccadic latencies for that latency bin (horizontal). The best performing participants showed spatiotopic curvature already at the shortest intersaccadic intervals. For the worst performing individuals it takes much longer before saccades start to curve away from the spatiotopic location. The grey line at the bottom represents the interval for which data was smoothed. (**b**). Difference in curvature between the smoothed data in the counterclockwise and clockwise conditions for the best performing participants (blue) and worst performing participants (orange) on the memory test. Shaded error bars represent the within-subjects SEM calculated over curvature. The blue horizontal line indicates significant clusters for the best performing individuals.

The absence of spatiotopic curvature for short intersaccadic intervals might have been due to the individual differences in memory maintenance. One might expect that successful updating is linked to better memory performance. To test this, we divided the participants in two groups depending on their performance on the memory test. A mixed-effects ANOVA on curvature difference (counterclockwise minus clockwise) with latency bin (1–4) as a within-subjects factor and memory performance group as a between-subjects factor revealed no main effect of latency bin (F(3,24) = 2.50, p = 0.08) or memory performance group (F(1,8) = 1.43, p = 0.27). However, there was a significant latency bin × memory performance group interaction (F(3,24) = 3.29, p = 0.04, η_p_^2^ = 0.29), indicating that the time-course was different between the two groups (see Fig. 4a). Analyzing both groups separately showed that updating was especially fast when memory performance was high. For these individuals there was no effect of intersaccadic interval on curvature (F(3,12) = 0.05, p = 0.98). In contrast, participants with poor memory performance showed an increase of curvature over time (F(3,12) = 4.36, p = 0.03, η_p_^2^ = 0.52).

The smoothed curvature difference time-course for both groups revealed a similar pattern (see Fig. 4). Updating was especially fast when memory performance was high. For these individuals saccades already curved away from the spatiotopic location of the memory cue when the intersaccadic interval was slightly longer than 100 ms. Cluster-based permutation testing confirmed this effect to be significant for intersaccadic latencies between 107 and 165 ms (*p* = 0.02). In contrast, for participants with poor memory performance spatiotopic curvature away slowly and steadily increased.

## Discussion

The results of Experiment 1 show that remembered locations are updated within the first 250 ms after an eye movement. This appears to be in line with earlier studies, demonstrating gradual updating of endogenously maintained information from retinotopic to spatiotopic coordinates^1,2,29^. Interestingly, the efficiency of updating differed substantially among participants and was related to performance on the memory test. Participants who performed well on the memory test demonstrated very fast updating of the memorized location. After little more than 100 ms saccades reliably curved away from the memorized location.

One possible explanation for this is the fact that two saccade targets were shown simultaneously, which allowed the preprograming of a saccade sequence. Preprograming allows for very short intersaccadic intervals^30^, but also makes the task more difficult. Besides actively maintaining the remembered location, participants also had to maintain and update the location of the second saccade target. Although some subjects were perfectly able to do so, for others this might have interfered with updating of the memorized location across the first saccade, resulting in both the absence of spatiotopic curvature away from the memorized location for the shortest intersaccadic intervals, and poorer performance on the memory test.

## Experiment 2

**Figure 5:**
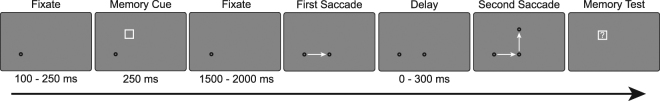
Experimental paradigm of Experiment 2. Participants fixated on the fixation dot and remembered the location of the cue. After a retention interval a saccade target appeared, and participants had to make a saccade. After a random interval between 0 and 300 ms after landing of the first saccade, another saccade target appeared and participants had to make a second saccade. At the end of the trial participants had to judge whether a test stimulus was presented at the same or at a slightly different location as the remembered cue.

To prevent preprogramming of saccade sequences in Experiment 2 both saccade targets were shown in succession (Fig. 5). Hence, the second saccade could only be prepared after the first saccade had been completed. The interval between landing of the first saccade and presentation of the second target was systematically manipulated, resulting in a broader distribution of intersaccadic intervals. The larger proportion of slower intersaccadic intervals allowed us to better examine the full time course of memory updating. If the relationship between memory performance and curvature in Experiment 1 was due to preprogramming of saccade sequence, the inability to do so in Experiment 2 should eliminate this relationship. Furthermore, in addition to fast updating of the memorized location, we expected its sustained maintenance over time.

### Methods

Fourteen participants, aged between 19 and 27 (mean 23, 9 female), participated in Experiment 2, consisting of two 75 minute experimental sessions, completing 576 trials. Experiment 2 was similar to Experiment 1 except that both saccade targets were shown in succession. The second saccade target appeared between 0 and 300 ms after landing of the first saccade. Afterwards the data were divided into four latency bins based on the total intersaccadic interval.

### Results

We used the same trial inclusion criteria as in Experiment 1. Two participants were excluded from further analysis because more than 60% of the experimental trials had to be excluded. For the remaining participants, there was an average loss of 14% of all trials. To ensure that participants were maintaining the spatiotopic location of the cue, memory performance was analyzed. All participants performed well on the memory test, with the average distance separating correct and false memory probes ranging between 2.50° and 4.48°. Performance on the memory test was not significantly different from Experiment 1 (2.93° versus 3.22°, t(20) = 1.12, p = 0.28; see Supplementary Fig. 1).

**Figure 6:**
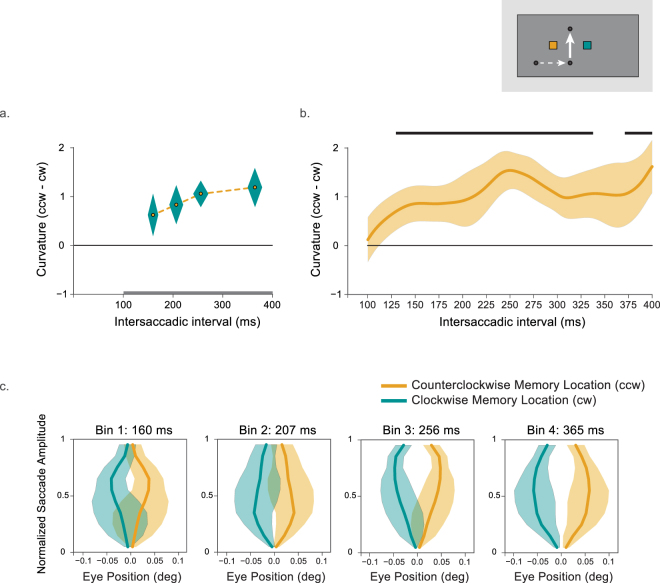
Results of Experiment 2. (**a**) Curvature difference for all four latency bins (counterclockwise minus clockwise memory location). The diamond shapes represent the within-subjects SEM calculated over curvature (vertical dimension) and the between-subjects SEM calculated over the mean intersaccadic latencies for that latency bin (horizontal). The grey line at the bottom represents the interval for which data was smoothed. (**b**) Average of the smoothed curvature of all participants (counterclockwise minus clockwise memory location). Shaded error bars indicate the within-subjects SEM calculated over curvature. The black horizontal lines indicate significant clusters. (**c**). Average saccade trajectories while memorizing locations at counterclockwise (orange) and clockwise (blue) locations for four latency bins.

For all four time bins, the difference in curvature between clockwise and counterclockwise conditions is plotted in Fig. 6a. Figure 6b shows the difference between these conditions as calculated by Gaussian smoothing. Figure 6c shows the normalized saccade trajectories in both the clockwise and counterclockwise condition. Overall, saccades curved away from the spatiotopic location of the memorized location (counterclockwise minus clockwise = 0.90°, one-tailed t-test: t(11) = 2.61, p = 0.01, Cohen’s d = 0.75). A repeated-measures one-way ANOVA on the curvature difference (counterclockwise minus clockwise) with latency bin (1–4) as a factor revealed no significant effect of latency bin on the degree of curvature (F(3,33) = 1.04, p = 0.39). Individual t-tests revealed that there was marginally significant curvature in the shortest latency bin (one-tailed t-test: t(11) = 1.43, p = 0.09), and significant curvature in the second (t(11) = 2.09, p = 0.03, Cohen’s d = 0.60), third (t(11) = 3.89, p = 0.001, Cohen’s d = 1.12), and fourth latency bin (t(11) = 2.96, p = 0.006, Cohen’s d = 0.86). Inspection of the smoothed data reveals that the eyes curve away from the spatiotopic location of the memory cue after intersaccadic intervals as short as 130 ms. For longer intervals the curvature difference between conditions remains at stable at around 1°. Cluster-based permutation testing confirmed this curvature difference to be significant for intersaccadic latencies between 130 and 336 ms and between 372 and 400 ms (*p* < 0.001).

**Figure 7:**
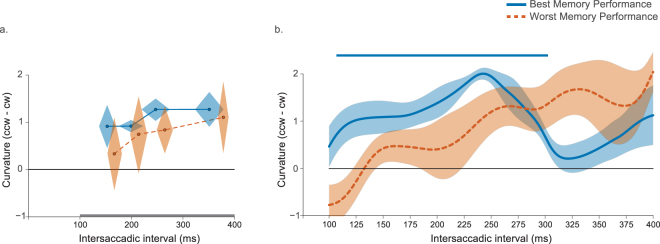
Saccade curvature as a function of memory performance in Experiment 2. (**a**) Calculated difference in curvature between counterclockwise and clockwise conditions for all four latency bins. Data from the best performing participants (blue) and worst performing participants (orange) on the memory test. The diamond shapes represent the within-subjects SEM calculated over curvature (vertical dimension) and the between-subjects SEM calculated over the mean intersaccadic latencies for that latency bin (horizontal). The grey line at the bottom represents the interval for which data was smoothed. (**b**) Difference in curvature between the smoothed data in the counterclockwise and clockwise conditions for the best performing participants (blue) and worst performing participants (orange) on the memory test. Shaded error bars represent the within-subjects SEM calculated over curvature. The blue horizontal line indicates significant clusters for the best performing individuals.

As in Experiment 1, we divided the participants in two groups depending on their memory performance. An ANOVA with latency bin (1–4) as within-subjects factor and memory performance group as between-subjects factor revealed no main effect of latency bin (F(3,30) = 0.96, p = 0.43) or memory performance group (F(1,10) = 0.26, p = 0.62). The interaction between latency bin and memory performance group was not significant (F(3,30) = 0.16, p = 0.92) (see Fig. 7). Despite the absence of an interaction effect, analysis of the smoothed data did reveal a pattern similar to Experiment 1. The best performing individuals already curved away from the spatiotopic location of the memory cue after very short intersaccadic intervals. Cluster-based permutation testing confirmed this effect to be significant for intersaccadic latencies between 108 and 301 ms (*p* < 0.001). For those performing worse on the memory test spatiotopic curvature increased slowly over time.

## Discussion

The results of Experiment 2 show that remembered locations were already updated within the first 130 ms after an eye movement. In addition, we showed that the spatiotopic representation remained stable over time, confirming that oculomotor competition reflects the continuous maintenance of a location in working memory.

The results show that the oculomotor competition was updated faster than in Experiment 1. This inconsistency can be explained by the inability to prepare two eye movements simultaneously. Note that in the first experiment both saccade targets were presented at the same time, which led to preprogrammed saccade sequences. In Experiment 2, the second target could only be processed after the execution of the first saccade, which rendered preplanning of such saccade sequences impossible. This facilitated saccades to the second target, which could now be made in an exogenous fashion. It might also explain why, in contrast to Experiment 1, analysis of the binned data did not reveal a relationship between memory performance and the efficiency of updating. Although spatiotopic curvature appeared to be stronger for the best performing individuals, the crucial interaction effect was absent. Analysis of the smoothed data does reveal a similar pattern as in Experiment 1: the best performing individuals already curved away from the spatiotopic location after little more than 100 ms, while this took longer for the individuals with the worst performance on the memory test.

## General Discussion

The present results demonstrate that locations of behaviorally relevant objects are rapidly updated across saccades. In Experiment 1 we showed that it takes more than 200 ms before the eyes curved away from the spatiotopic location of a memorized cue after an intervening eye movement. In Experiment 2, when preprogrammed saccade sequences were impossible, updating was even faster. The eyes curved away from the spatiotopic location of a memorized cue within 130 ms after the first eye movement. In addition, we showed that the time-course of updating spatial information depended on how well the location was memorized. Updating was especially fast when memory performance was high. In addition, we showed that the spatiotopic representation remains stable over time.

The results are inconsistent with current models of updating working memory during eye movements. Previous studies suggest that the transformation of a memory representation into spatiotopic coordinates is a time-consuming and effortful process, taking up to 400 ms^1,2,29^. This led to the hypothesis that the native coordinate system of memory representations is retinotopic; memorized locations naturally move along with each eye movement and a special effort is needed to transform these retinotopic representations into the world-centered coordinates^1,2^. Here we demonstrate that memorized locations are updated much faster. Despite the absence of any landmark objects or visual references which might help updating, this transformation was completed within the first 130 ms following an eye movement. One could argue that earlier studies are equally consistent with such a fast remapping of activity. In some experiments relatively early spatiotopic effects were found^1^. However, these effects were always accompanied by a retinotopic trace that was just as strong or stronger. In the present paradigm curvature away from the spatiotopic location can only emerge if the spatiotopic activity dominates over the retinotopic trace, suggesting that significant unmapping of the retinotopic trace had already taken place.

The rapid emergence of spatiotopic memory representations observed here is comparable to the updating of exogenous attentional signals^8–10^. In these tasks nothing needed to be memorized, but instead an irrelevant but salient cue was used to capture attention. When this cue was flashed shortly before saccade execution, attention resided at its spatiotopic location directly afterwards. Furthermore, studies of saccadic IOR have reported similar rapidly emerging spatiotopic representations^12–14^ (but see ref.^16^). The study that is most comparable to ours used saccade curvature to show that spatiotopic representations also emerge rapidly and automatically in the oculomotor system^19^. Saccades curved away from the spatiotopic location of an attended location after a similar interval as we show here for endogenously maintained locations, indicating that a single mechanism might be involved in the updating of relevant locations, independent of whether these locations are prioritized in an endogenous or exogenous manner.

Although both memorized locations and exogenous attentional signals are updated in a similar fashion, there are some crucial differences between both tasks. Where memory involves the maintenance of a stable representation over a longer period of time, exogenously triggered shifts of attention are transient events. The location of exogenous cue was updated only when it captured attention directly before the start of an eye movement and its attentional facilitation lasted only briefly after saccade landing (e.g. ref.^8^). In contrast, when maintaining a location in memory its representation has to remain stable after updating has been completed. Therefore, if the oculomotor activity we measured indeed reflects memory maintenance, saccades should still curve away from the spatiotopic location long after the saccade. The results of Experiment 2 show that this was indeed the case. In contrast to exogenous attention, updated activity did not decay over time but remained stable for at least 400 ms.

Performance on the memory test appears to be related to the efficiency of updating. Differences between participants were most pronounced for the fastest eye movements. Participants that were better able to accurately indicate the memorized location at the end of a trial also showed strong spatiotopic curvature after short intersaccadic intervals. In contrast, saccades of poorer performing participants did not curve away from the spatiotopic location unless this interval was at least 200 ms. One possible explanation for this finding is that the saccade task interfered with updating when participants tried to make very fast saccade sequences. Such sequences require either preprogramming or extremely fast preparation of the second eye movement. In Experiment 1, participants could preprogram a saccade sequence. Preprogramming of such sequences involves the simultaneous preparation of two saccades (see for example^31^). Although this enables very short intersaccadic intervals, it also necessitates rapid updating of the location of the second saccade target across the first eye movement^30^. This might have interfered with the updating of the memorized location. Some participants might not have been able to update two locations at the same time, resulting in the absence of spatiotopic curvature after preprogrammed saccade sequences. Others did show spatiotopic curvature after these extremely fast saccade sequences, demonstrating that there are individual differences in the ability to simultaneously update two locations. In Experiment 2, preplanning of a saccade sequences was impossible; the second saccade target only appeared after the first saccade had been executed. Still, participants were able to make very fast saccade sequences and we found a similar (although slightly weaker) relationship between memory performance and updating.

Efficient updating of spatial memory has been shown to depend on the continuous presence of reference objects. In a study by Lisi and colleagues^29^ participants had to maintain either an empty location in space or one of several locations indicated by a placeholder object. When attention had to be directed to an empty location in space, only a retinotopic trace was observed after an intervening eye movement. However, the presence of placeholder objects significantly changed the time-course of updating and resulted in spatiotopic facilitation shortly after a saccade. Golomb and colleagues^7^ obtained similar results when they added a static background frame to their paradigm. In the current paradigm, there were neither placeholders nor continuously present landmarks which could have helped updating. Except for the second saccade target the paradigm was very similar to that of Golomb and colleagues^1,7^. Still, the time-course of updating of the memorized location was considerably faster than demonstrated in the previous experiments. Although landmark objects might aid in the precise localization of objects across saccades, the present findings suggest that they are not a necessary requirement for fast updating. This is in line with previous work suggesting that it takes some time before the locations of landmark objects are processed after an eye movement^17^. In this study participants had to memorize the location of a target across a saccade. Although a fast spatiotopic representation was already available shortly after saccade landing, the location of reference objects was shown to have little effect on localization unless there was enough time to process new retinal input.

It has been proposed that maintenance of accurate spatial representations across saccades is especially important for actions, but is less critical for perception^32^. It is not necessary to have a precise prediction of the post-saccadic location of a continuously present object because it can easily be relocated upon saccade landing. However, in order to interact with objects and to avoid obstacles, the updating of potential movement goals is crucial. Moreover, this updating has to be fast. Even when doing a simple daily task as brewing a cup of coffee we make multiple eye movements per second. Having to gradually update our movement goals after each of these eye movements would render such task impossible. In line with this, it has been hypothesized that spatial attention is generated in the same neural circuits used to plan and execute motor actions^33,34^. According to this idea any shift of attention is a consequence of preparation of an eye movement^35,36^. Given the dominant role of the motor system in representing spatial information, saccade curvature might be the ideal tool to measure the updating of these representations. It allows us to directly and unobtrusively tap into the oculomotor map of the SC, and does not require any additional perceptual task.

There are several mechanisms that could mediate rapid formation of spatiotopic representations in the oculomotor system. One theory describes the predictive updating of attentional pointers^11^. These pointers indicate task-relevant locations in spatial priority maps. Each time a saccade is prepared a copy of its motor command is used to predictively update this pointer, thereby bridging the spatial discrepancies between successive fixations and enabling us to efficiently update prioritized locations or movement goals across saccades. The predictive updating of exogenous attention that was recently demonstrated is in line with this idea^8–10^. Priority maps do not merely code for bottom-up saliency, but locations can also be prioritized based on task demands. Given the close link between attention and spatial working memory^3,5^, maintenance of a location in memory is likely to rely on updating of attentional pointers.

The idea that the maintenance of a location relies on the updating of a motor plan might also, at least to some extent, explain the discrepancy of our results with previous studies. Most researchers used perceptual discrimination tasks to investigate updating of memorized content, which are thought to depend on attentional modulations in visual cortex. The efference copy driving remapping is likely to be generated in the SC, one of the latest stages along the sensorimotor continuum, and subsequently fed back to cortical areas earlier in the visual system through the thalamus^37–39^. Neurophysiological studies suggest that remapping is observed later in time for the areas earlier in visual hierarchy^40–45^, which makes it likely that there is some delay between updating oculomotor competition within the SC and the emergence of perceptual enhancement at the corresponding location in visual cortex.

Taken together, we show that target-distractor competition as a result of remembering a location is rapidly (within the first 130 ms) updated into spatiotopic coordinates after an intervening eye movement. The time-course is comparable to the updating of exogenous attention^19^ and is inconsistent with the view that postulates a gradual deliberate shift from retinotopic to spatiotopic memory representations.

### Data availability statement

The datasets generated during and/or analyzed during the current study are available in the Open Science Framework repository; osf.io/25b6s.

## Electronic supplementary material

Supplementary Material
