# Supplementary material for: Rapid updating of spatial working memory across saccades
Source: Sci Rep. 2018 Jan 18;8:1072. doi: 10.1038/s41598-017-18779-9 (PMC5773520; doi:10.1038/s41598-017-18779-9)
Supplement: Supplementary file 1 — Supplementary Material [file 41598_2017_18779_MOESM1_ESM.pdf]

Supplementary Material for: Rapid updating of spatial working memory across saccades.

Paul J. Boon, Silvia Zeni, Jan Theeuwes, Artem V. Belopolsky

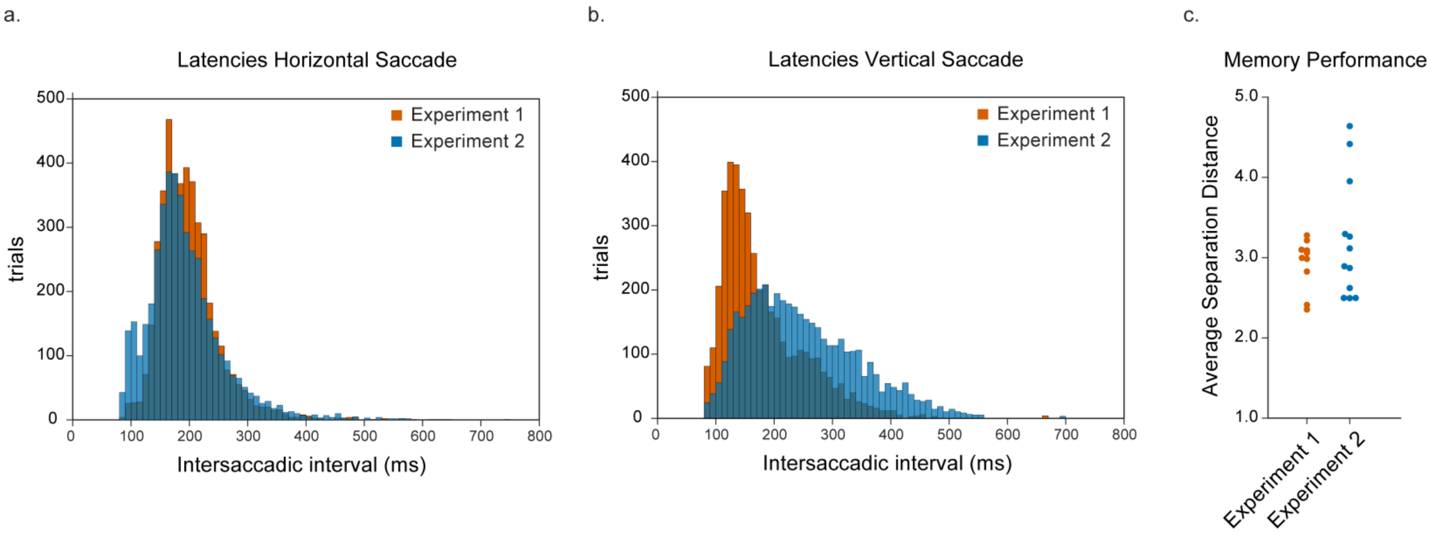

Supplementary Figure 1: a. Histogram of latencies of the first (horizontal) saccade for both Experiment 1 (orange) and Experiment 2 (blue). b. Histogram of latencies of the second (vertical) saccade for both Experiment 1 (orange) and Experiment 2 (blue). c. Average distance between correct and incorrect memory probe for all participants in Experiment 1 (orange) and Experiment 2 (blue). The separation distance was continuously adjusted during the experiments by a staircase procedure, aiming at keeping performance at 75% correct.
